# Diversity and Succession of Bacterial Communities in the Uterine Fluid of Postpartum Metritic, Endometritic and Healthy Dairy Cows

**DOI:** 10.1371/journal.pone.0053048

**Published:** 2012-12-27

**Authors:** Thiago M. A. Santos, Rodrigo C. Bicalho

**Affiliations:** Department of Population Medicine and Diagnostic Sciences, College of Veterinary Medicine, Cornell University, Ithaca, New York, United States of America; Institute for Genome Sciences, University of Maryland School of Medicine, United States of America

## Abstract

The diversity of the uterine bacterial composition in dairy cows is still poorly understood, although the emerging picture has shown to be increasingly complex. Understanding the complexity and ecology of microorganisms in the uterus of postpartum dairy cows is critical for developing strategies to block their action in reproductive disorders, such as metritis/endometritis. Here, we used PCR-Denaturing Gradient Gel Electrophoresis (DGGE) and DNA pyrosequencing to provide a comprehensive description of the uterine bacterial diversity and compare its succession in healthy, metritic and endometritic Holstein dairy cows at three intervals following calving. Samples were collected from 16 dairy cows housed in a dairy farm located in upstate New York. PCR-DGGE revealed a complex profile with extensive differences in the community structure. With few exceptions, clustering analysis grouped samples from cows presenting the same health status. Analysis of >65,000 high-quality 16S rRNA gene sequences showed that the uterine bacterial consortia, regardless of the health status, is mainly composed of members of the phyla Bacteroidetes, Fusobacteria, Firmicutes, Proteobacteria, and Tenericutes. In addition to these co-dominant phyla, sequences from Spirochaetes, Synergistetes, and Actinobacteria appear less frequently. It is possible that some sequences detected in the uterine fluid resulted from the presence of fecal or vaginal contaminants. Overall, the bacterial core community was different in uterine fluid of healthy cows, when compared to cows suffering from postpartum diseases, and the phylogenetic diversity in all the combined samples changed gradually over time. Particularly at the 34–36 days postpartum (DPP), the core community seemed to be specific for each health status. Our finding reveals that the uterine microbiota in dairy cows varies according with health status and DPP. Also, it adds further support to the hypothesis that there is uterine contamination with diverse bacterial groups following calving and emphasizes the role of unidentified microorganisms in this context.

## Introduction

Bovine postpartum diseases, such as metritis and endometritis, remain one of the largest costs to the dairy industry [1], [2] and it has long been known that bacterial contamination of the uterine lumen following parturition is the major cause of such disorders [3], [4]. Most evidence to support the bacterial role in the pathogenesis of these uterine infections has been provided by culture-dependent studies that isolated numerous pathogens, such as *Escherichia coli*, *Arcanobacterium pyogenes*, *Fusobacterium necrophorum*, *Prevotella melaninogenicus*, *Bacteroidetes* spp., *Pseudomonas* spp., *Streptococcus* spp., and *Staphylococcus* spp., in a variety of combinations, from cows diagnosed with postpartum metritis [5]–[9].

Knowing the diversity of the uterine microbiota may provide additional insight into the ecology of species related to reproductive disorders and is critical for efficient therapeutic interventions. However, the task of clarifying the microbial composition involved in the etiology of metritis and endometritis by traditional methods might be seriously hampered by the fact that more than 99% of the microorganisms present in the environment are not amenable to cultivation under standard laboratory conditions [10], [11]. In fact, recent nucleic acid-based studies by our group showed that the diversity of the uterine bacterial composition in healthy dairy cows and in cows suffering from puerperal metritis is likely to be even more complex than previously known [12].

In the present report, we used high-throughput automated DNA pyrosequencing to perform a comprehensive description of the bacterial core community in the uterine fluid of healthy Holstein dairy cows and cows suffering from puerperal metritis and/or endometritis. Our eventual aim is to expand the current picture of the bacterial composition of the bovine uterine microbiome and find out whether a particular community structure correlates with metritis and endometritis, which could ultimately facilitate disease prediction and even suggest possible therapeutic entry points for bovine reproductive disorders.

## Results

### Analysis of the uterine bacterial composition by PCR-Denaturing Gradient Gel Electrophoresis (DGGE)

**Figure 1:**
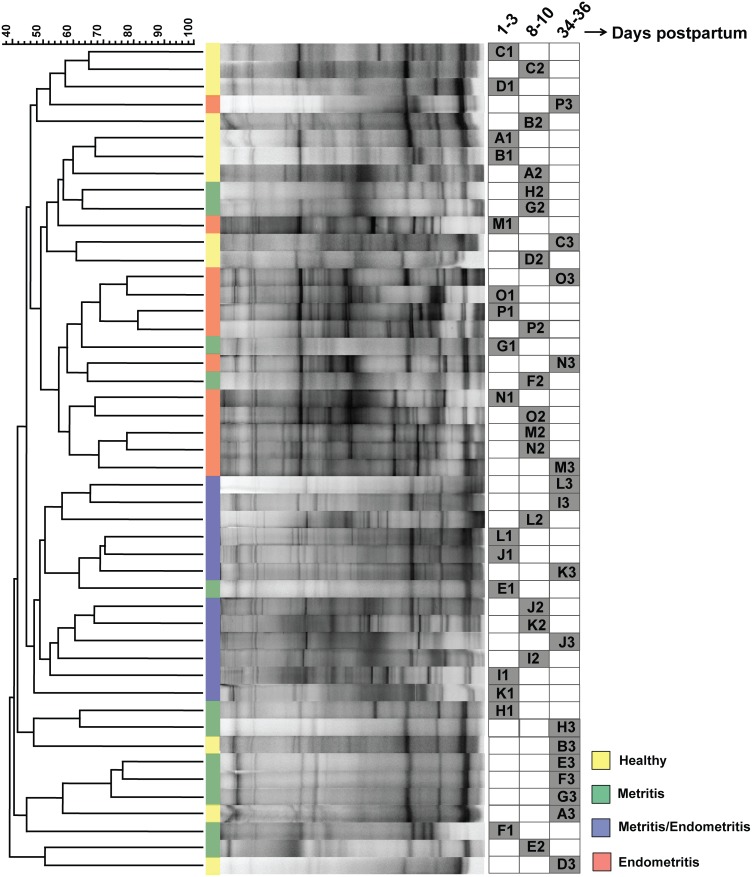
DGGE fingerprinting and clustering analysis of banding profile of the amplified bacterial 16S rRNA gene fragments from total genomic DNA extracted from uterine fluid of dairy cows. The dendrogram illustrating the similarities among the bacterial profiles in different cows with different health status along different days postpartum (DPP) were calculated based on the intensity and the position of each band in the the gel. The resulting patterns were compared to one another using the Dice similarity coefficient, and the matrix was clustered by the UPGMA method. Scale bar indicates percentage of similarity. The vertical line between the terminal branches in the dendrogram and the DGGE profiling are color-coded according to the clinical status of the cow. The matrix on the right (filled boxes) indicates the sampling date (as DPP following parturition). Identical letters refer to the same cow in one of the 3 DPP intervals of sampling (A–D, healthy; E–H, metritis; I–L, metritis/endometritis; M–P, endometritis). The number following the letter refer to the sampling interval for that specific dairy cow. In all cases, clustering was supported by high cophenetic correlation coefficients (omitted for clarity).

Profiling of the bacterial diversity by using two-step nested PCR-DGGE revealed differences in the structure of the communities present in all the samples (Figure 1). The number of bands (i.e., phylotypes or Operational Taxonomic Units, OTUs) on the gel, which is an approximate indicative of richness, revealed a complex profile. The fingerprint obtained was used to compare the bacterial diversity in uterine fluid samples from dairy cows at different days postpartum (DPP) immediately after parturition. The cluster analysis showed that, with few exceptions, the profiles from cows presenting the same health status grouped together in separate clusters (Figure 1). Overall, there was a distinct separation based on the health status of the cows. Particularly, this was more obvious clustering of samples from cows that were diagnosed with endometritis and endometritis followed by metritis. In contrast, no consistent organization was observed when DPP was compared. It seemed evident that clustering by PCR-DGGE was dictated by the health status of the animal. In all cases, clustering was supported by high cophenetic correlation coefficients.

### Diversity of uterine bacterial phylotypes as evaluated by DNA pyrosequencing

**Figure 2:**
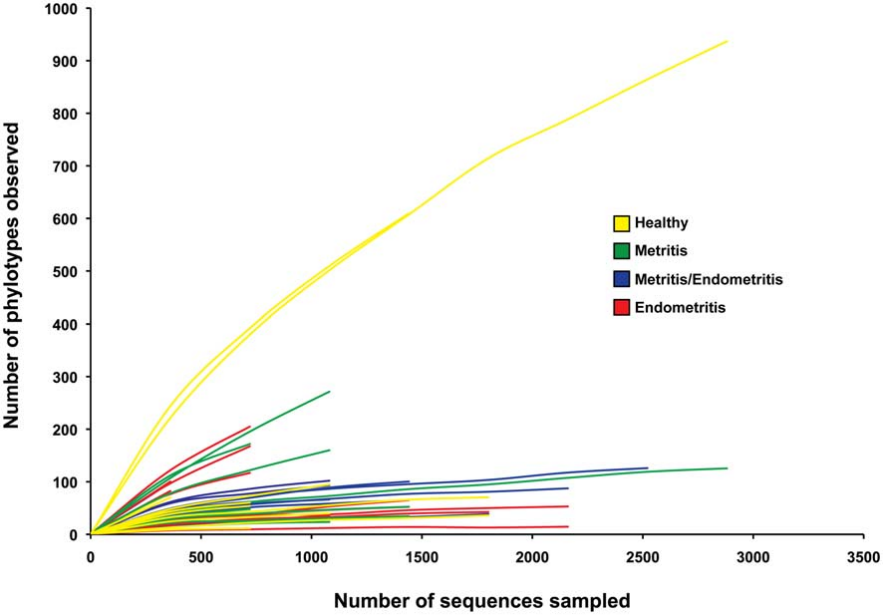
Rarefaction analysis of the 16S rRNA gene fragment data from bovine uterine sample indicating the operational taxonomic unit (OTU) richness. Individual rarefaction curves for each uterine sample taken. All the sequences with less than 3% divergence from one another were considered the same OTU.

We were able to obtain a combined total of 65,376 high-quality 16S rRNA sequences reads. The sequences obtained were assigned to specie-level OTUs using 97% pairwise identity cutoff and a total of 2,933 different OTUs were detected in the 48 different samples. Coverage values were >90% for 75% of the samples and ≥95% for 67% of the samples (Table 1). Rarefaction analysis showed that, for some samples, the number of sequences screened was sufficient to reveal the total number of sequence types within these samples, as they reached a plateau (Figure 2). For other samples, the rarefaction curves showed that more sequences would be necessary to capture all sequence types present in order to reflect the complete diversity within the sample. Interestingly, the two samples that showed the steepest rarefaction curve (samples B3 and C3, Table 1) were the ones with the highest number of sequences sampled. This suggests that these two samples were the most diverse among the 48 different samples. Both were sampled from healthy dairy cows (Table 1 and Figure 2). The large number of phylotypes found in both samples suggested that their microbiota was more diverse than that found in all the other samples, as confirmed by the diversity indexes. Both samples presented the highest diversity indexes (Table 1), so this observation was not a simple artifact of the sequencing method or the variations in the number of sequences screened for each sample.

**Table 1:** Sequence diversity and coverage estimations for bacterial 16S rRNA gene analysis from uterine samples.

Health status	Cow ID	Number of phylotypes^a^	H′^b^	Chao1^c^	Singletons	Number of sequences^d^	% Coverage^e^
Healthy	A1	357	4.99	803.17	228	3533	93.5
	A2	57	3.81	107.60	23	832	97.2
	A3	102	4.47	161.91	53	676	92.2
	B1	72	3.11	131.50	35	1852	98.1
	B2	63	3.35	133.13	34	780	95.6
	B3	946	8.37	1796.20	546	2936	81.4
	C1	38	1.47	130.00	24	1994	98.8
	C2	49	3.48	119.86	32	289	88.9
	C3	646	8.56	1230.38	375	1599	76.5
	D1	12	1.00	17.00	5	1014	99.5
	D2	41	3.29	69.88	22	1155	98.1
	D3	98	3.84	216.07	58	1171	95.0
Metritis	E1	52	3.26	145.50	34	829	95.9
	E2	71	1.60	224.11	53	1617	96.7
	E3	206	5.32	610.46	151	723	79.1
	F1	38	2.86	72.00	17	1715	99.0
	F2	131	3.90	439.10	79	3093	97.4
	F3	234	4.96	833.08	177	1043	83.0
	G1	50	3.65	83.00	22	836	97.4
	G2	53	3.30	91.00	20	1550	98.7
	G3	107	4.46	266.75	72	559	87.1
	H1	297	5.08	1044.79	226	1216	81.4
	H2	27	0.85	45.20	14	1307	98.9
	H3	171	4.87	410.80	110	1235	91.1
Metritis/Endometritis	I1	77	3.52	152.25	43	1364	96.8
	I2	118	5.07	273.55	59	1368	95.7
	I3	101	4.82	175.10	39	1305	97.0
	J1	68	3.13	126.00	29	1587	98.2
	J2	104	4.51	174.83	51	1594	96.8
	J3	23	2.05	41.33	11	595	98.2
	K1	40	1.93	65.50	18	2117	99.1
	K2	70	4.08	115.09	32	853	96.2
	K3	33	2.63	68.00	15	1257	98.8
	L1	92	4.10	136.00	33	2471	98.7
	L2	129	4.24	225.25	56	2742	98.0
	L3	6	1.28	6.50	2	262	99.2
Endometritis	M1	113	5.39	290.50	71	449	84.2
	M2	49	2.93	74.67	22	2160	99.0
	M3	131	4.93	277.71	79	718	89.0
	N1	196	5.23	504.79	132	900	85.3
	N2	137	4.77	349.69	83	982	91.5
	N3	54	3.15	99.11	29	1495	98.1
	O1	15	0.87	43.00	8	2347	99.7
	O2	56	2.46	93.50	25	2506	99.0
	O3	52	4.70	134.67	32	182	82.4
	P1	105	4.79	343.91	73	385	81.0
	P2	27	1.95	31.00	8	911	99.1
	P3	43	1.72	131.00	33	1272	97.4

a Number of operational taxonomic units (OTUs) found within each sample.

b H′ (Shannon-Weaver Index of diversity) indicates species richness, considering the abundance of individual taxa. A higher number indicates more diversity.

c Chao1 is a non-parametric estimator used to predict species richness (the total number of OTUs present) in each sample based on distribution of singletons and doubletons.

d Number of sequences for used for comparative analysis.

e %Coverage is calculated to estimate the representation of the phylotypes in the samples.

The Chao1 non-parametric estimator, used to predict species richness in each sample based on the distribution of singletons and doubletons, predicted about 1,796 OTUs in sample 44 (from a healthy dairy cow) and 6 OTUs in sample 14 (from a cow diagnosed with endometritis following metritis after 34–36 DPP); the maximum and minimum values predicted. In general, the samples from healthy cows and from cows suffering from metritis were predicted to have the highest numbers of OTUs (Table 1), according to this estimator. In addition, because the current sequencing effort was not sufficient to reach a plateau for some of these samples, these numbers are likely to be a minimal estimate. The highest value for the measure of diversity (Shannon index) was also observed for the libraries from healthy dairy cows, followed by the libraries from cows suffering from metritis (Table 1).

### Composition and succession of the bacterial consortia in the uterine fluid

The final complete data set included representatives of, at least, 19 bacterial phyla. Eleven groups were present in less than 0.1% (together they represented only 2.8% of the total sequences) and, for clarity, were combined and named “Other”. This systematic group also encompassed some sequences whose affiliation assignment was not clear. It is possible that these sequences with unclear affiliation belong to a new group of bacteria uncultured or unidentified thus far.

**Figure 3:**
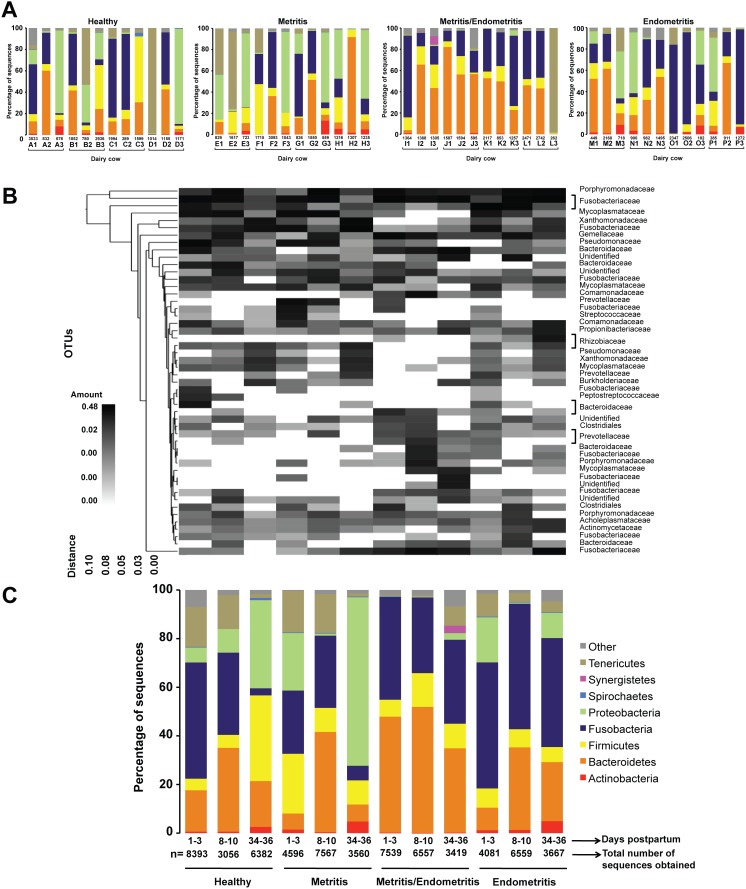
Operational taxonomic unit (OTU)-based community structure and composition of the uterine bacterial consortia in dairy cows. (A) Stacked bars showing the relative abundance and succession of each bacterial phylum in uterine fluid of dairy cows over the days across individual dairy cows over the days. Numbers below the bars refer to the total number of the sequences obtained for each cow and letters followed by numbers refer to the dairy cow identification and sampling interval (days postpartum, DPP), respectively. For the color code, refer to the key in panel 3C. (B) Each vertical lane on the heat map corresponds to a combination of 16S rRNA gene fragments from samples obtained from the 4 dairy cows presenting the same health status in one of the 3 DPP following parturition. The gray-scale heat map represents the relative abundance of the 50 most occurring OTUs. Taxonomic identification at the family level is indicated on the right. The dendogram on the left represents the OTUs clustering and provides the family designation along the right y-axis and the abundance relationship across all samples for each family based upon UPGMA clustering and Manhattan distance methods along the left y-axis. The relative distance scale for the left y-axis is provided in the lower left corner of the figure. (C) Stacked bars showing the combined abundance and succession of each bacterial phyla in uterine fluid shared by all dairy cows over the days. For the analysis shown in C, the data obtained from the three different cows were grouped based on health status and days postpartum.

Taxonomic assignment showed that the dominant uterine bacterial phyla were Fusobacteria, Bacteroidetes, Proteobacteria, Firmicutes, Tenericutes. Sequences from Spirochaetes, Synergistetes, and Actinobacteria are also present, but less frequently and only in some samples. Examination of individual OTU diversity at phylum level across all samples showed noticeable differences between individual cows reflected manly by changes in the abundance of the dominant groups (Figure 3A). Particularly, the distribution of groups in healthy cows and in cows diagnosed with metritis varied abruptly over the days and no obvious shifting patterns was observed for most cows. In both categories, even though OTUs affiliated with Fusobacteria appeared frequently, this group of bacteria does not represent a large fraction of the diversity at the 34–36 DPP. At least for cows with metritis, Proteobacteria was the dominant phylum at the 34–36 DPP in all the cows used in the study. For cows that were diagnosed with metritis followed by endometritis, the core bacterial community shared by all cows was more similar between individual cows and shifted more predictably, except for cow L, in which the three major groups found in this category (Bacteroidetes, Firmicutes, and Fusobacteria) were surprisingly outnumbered by Tenericutes at the 34–36 DPP. Proteobacteria was a minor phylum in this category and, in some cases, was not even found. In cows with endometritis, Bacteroidetes were identified in high numbers during the 1–3 and 8–10 DPP, but were outnumbered by other groups, particularly Proteobacteria and Fusobacteria, at the 34–36 DPP.

The abundance of OTUs was additionally grouped according to health status and the DPP to facilitate comparative analyses between the various conditions throughout the course of the study (Figure 3B and 3C). The OTUs were clustered in a heat map according to their co-occurrence (Figure 3B). This analysis included only the 50 most occurring-frequent OTUs and described the distribution and succession of the OTUs at the family-level. This clustering analysis did not reveal a clear succession of bacterial communities in discrete phases according to health status or DPP. In the combined analysis, the majority of the sequences (about 97.2% of the total high-quality sequences reads) were classified as Fusobacteria (34.3%), Bacteroidetes (29.1%), Proteobacteria (12.5%), Firmicutes (12%), Tenericutes (7.7%), Actinobacteria (1.3%), Synergistetes (0.2%), and Spirochaetes (0.1%). Overall, there was an unpredictable shift of the microbiota composition in all the health statuses over time, suggesting that the bacterial community is dynamic and that bacterial succession might be occurring (Figure 3C).

The samples from uterine secretion from healthy cows contained diverse phylotypes mainly affiliated with Bacteroidetes, Firmicutes, Fusobacteria, Proteobacteria, and Tenericutes (Figure 3C). Analysis of the combination of individual libraries from the 4 healthy animals sampled at the 1–3 DPP showed that Fusobacteria dominated the sequence collection and represented more than 47% of the sequences. Fusobacteria was followed by Bacteroidetes and Tenericutes, which represented 17% and 16.4%, respectively. At the 8–10 DPP, the initial structure observed for the community was maintained. However, there was an increase (about 100%) in Bacteroidetes and concomitant decrease in Fusobacteria. Even more dramatic was the shift observed at the 34–36 DPP, in which Fusobacteria is reduced to less than 5% of the total number of sequences and the number of Bacteroidetes returned to its initial level, observed in the beginning of the course of the study for this group of cows. Additional shift was observed in the numbers of Firmicutes, which increases from 5.3% of the population present at the 8–10 DPP to 35.3% of the population at 34–36 DPP. Similar increase was also observed for the population of Proteobacteria, which increased from about 10% (8–10 DPP) to more than 36% (34–36 DPP).

The phylotypes occurring in the samples from uterine secretion from cows that had metritis contained phylotypes affiliated mainly with Bacteroidetes, Firmicutes, Fusobacteria, Proteobacteria, and Tenericutes (Figure 3C). Interestingly, these were the same phyla present in sequences from healthy cows. However, the distribution and succession of these phyla throughout the days were completely different of that observed for samples from healthy cows; the 5 major taxonomic groups present in these samples showed abrupt and random shifts in abundance over time. Initially, at the 1–3 DPP, Firmicutes, Fusobacteria, Proteobacteria were evenly distributed and each of them represented about 24% to 26% of the total population. Following, Tenericutes and Bacteroidetes represented 17% and 6.6%, respectively. At the 8–10 DPP, while the percentage of sequences affiliated with Fusobacteria and Tenericutes remained almost the same, the number of Bacteroidetes increased to more than 41% of the total population. Concomitantly, there was a substantial reduction in the numbers of Firmicutes to about 10% of the total. Proteobacteria was almost absent, representing about 1% of the population. Surprisingly, at the 34–36 DPP, the population of Proteobacteria outnumbered all the groups and accounted for almost 70% of the total population (Figure 3C). In general, the dynamic of the bacterial population was more chaotic and showed abrupt variations when compared to that of the other health statuses.

In the pool of sequences from samples obtained from dairy cows that developed endometritis following metritis, most sequences were affiliated with Bacteroidetes, Firmicutes, and Fusobacteria (Figure 3C). Bacteroidetes and Fusobacteria dominated the sequence collection. Particularly, at the 1–3 and 8–10 DPP, these two groups together represented 90% and 83% the sequence collection, respectively. At the 34–36 DPP, a small decrease in the number of OTUs was observed for both groups and three other groups (Tenericutes, Synergistetes, and Proteobacteria) had a slight increase. Overall, these three combined samples had the most homogeneous pattern and static population over time. Similarly, the overall distribution and succession of the OTUs in samples from animals that developed endometritis independently of previous development of metritis had a less impressive shift. The number of sequences phylogenetically affiliated with Fusobacteria and Firmicutes remained almost the same throughout the course of the study, varying from about 50% to 45% and 8% to 6% of the total, respectively (Figure 3C). Proteobacteria, which represented approximately 18% of the total at the first day of sampling (i.e., 1–3 DPP), dropped to less than 1% at the 8–10 DPP and increased again to almost 11% of the total population at the 34–36 DPP. Bacteroidetes, which initially represented less than 8% of the population, rapidly increased to represent about 34% of the total sequences at the 8–10 DPP and finally dropped to 24%.

**Figure 4:**
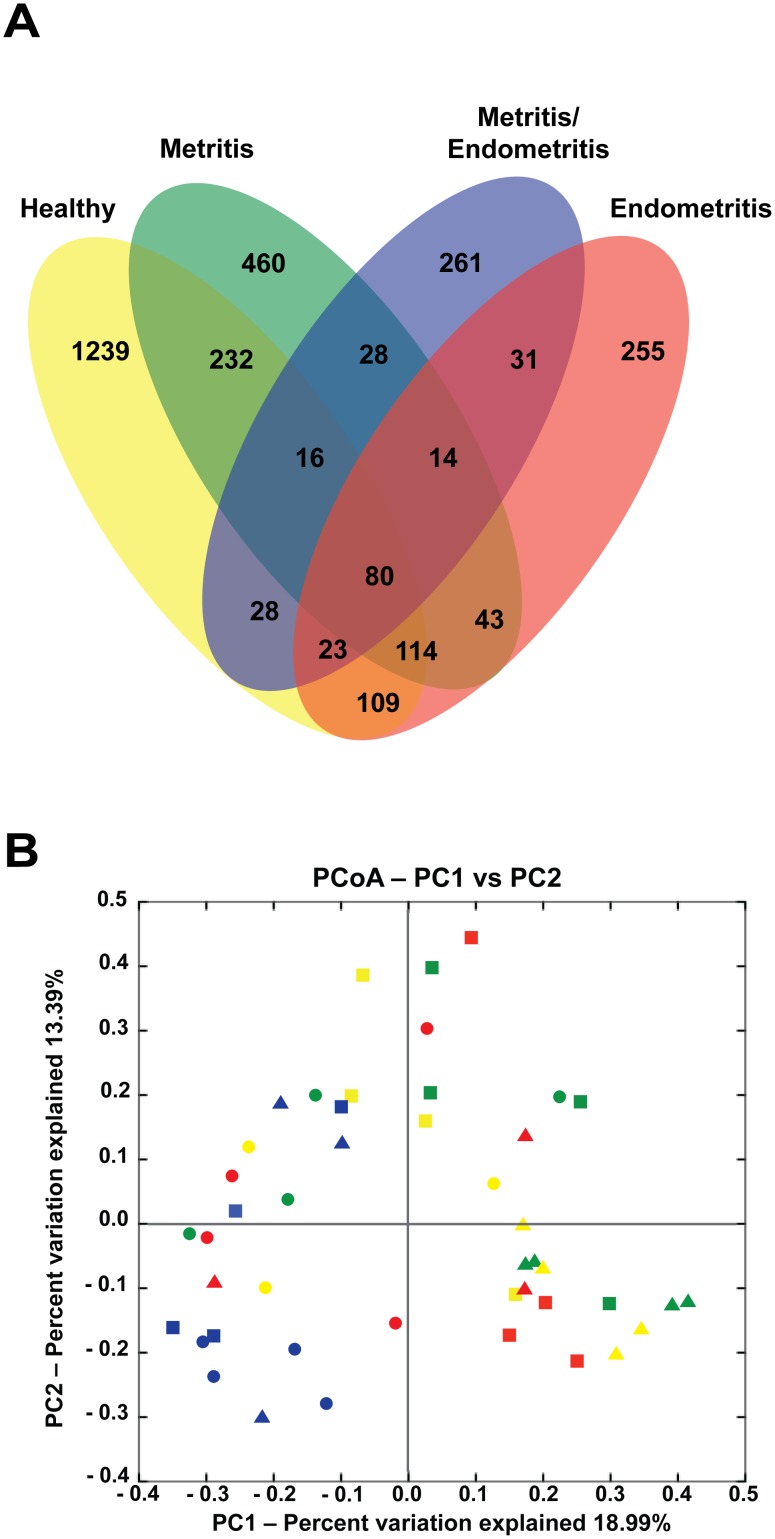
Association between the uterine bacterial consortia composition in uterine fluid and the host health status. (A) Description of the number of OTUs specific for each health status or shared between different health statuses, represented in a Venn diagram. OTUs were defined at 3% distance level. (B) Principal component analysis (PCoA) of UniFrac unweighted distance metric between bacterial communities determined from 16S rRNA genes. Percentage variation explained by each PCoA is indicated on the axis. Each symbol represents an individual sample obtained from dairy cows presenting different health status (H, healthy [yellow]; M, metritis [green]; M/E, metritis/endometritis [blue]; and E, endometritis [red]) in one of the 3 DPP following parturition (1–3, squares; 8–10, circles; 34–36, triangles).

About 2.7% (80 OTUs) of the total 2,993 different OTUs detected are common to all the 4 health statuses under investigation in the present study (Figure 4A). Surprisingly, more than 41% of this total appeared exclusively in samples collected from healthy cows. Disease-associated OTUs (i.e., OTUs exclusively appearing in cows with metritis, metritis/endometritis, or endometritis) represented 36.5% of the total. About 1.3% of the disease-associated OTUs were common to all disease statuses. Principal component analysis (PCoA) of overall diversity based on UniFrac (unweighted) metric showed that, in general, even samples from cows with the same healthy status and at the same DPP presented different bacterial community (Figure 4B). However, we observed that the majority of samples from cows with metritis/endometritis (Figure 4B, blue squares) grouped, suggesting that they were more similar to each other as opposed to samples from other health statuses. This observation was in agreement with the analysis of relative abundance of the bacterial phyla described above (Figure 3A and 3C).

## Discussion

Recent study from our group has suggested that the diversity of the uterine bacterial composition in dairy cows is likely to be even more complex than previously described by traditional culture-dependent methods [12]. Surveys of bacterial populations based on high-throughput automated DNA pyrosequencing of 16S rRNA gene sequences are revealing the staggering and previously unappreciated complexity of the microbiota that resides in a variety of different habitats inside the host's body [13]–[15]. In the present study, we combined PCR-DGGE and 454 pyrosequencing to characterize the uterine bacterial diversity of dairy cows with different health statuses in order to better understand the etiology of reproductive disorders in bovine cattle. Our data provide comprehensive insights into the current picture of the uterine bacteriome in healthy dairy cows and dairy cows with metritis and/or endometritis. To our knowledge, the present study represents the first comparative phylogenetic characterization of the diversity and succession of the bacterial biota in uterine fluid of dairy cows by using 454 pyrosequencing.

The richness and diversity of OTUs identified in our study surpassed that described in previous studies based on traditional cultural methods [5], [16] or in our previous culture-independent screening [12], in which the number of clones analyzed (272 clones total) was greatly inferior to the number of sequences described here. Both approaches combined allowed us to detect even bacteria that otherwise might be fastidious, obligate intracellular, or noncultivable. Most high-quality sequence reads obtained, regardless of the health status of the cow, belonged to the phyla Bacteroidetes, Fusobacteria, Firmicutes, Proteobacteria, and Tenericutes. The co-occurrence and dominance of these phyla also adds further support to our previous study [12] and to the hypothesis that metritis and endometritis are resultant of uterus contamination with diverse bacterial groups [5], [17]. In particular, it emphasizes the determinant role of specific groups in this context. Surprisingly, the clustering analysis suggested that the bacterial community assembly in samples from cows suffering from metritis was more closely related to samples from healthy cows than to samples from cows with endometritis or metritis/endometritis. However, it is clear that the phylogentic composition is shaped differently in cows suffering from metritis, in which chaotic shifts in the bacterial consortia were observed over time.

Classically, *E. coli* and *A. pyogenes* have been acknowledged as critical players in the establishment of metritis [9], [18], [19]. In particular, it has been long suggested that uterine infections might depend on pathogenic synergism between these two bacteria and bacteria from other groups [20]. For example, *E. coli*, commonly isolated during the first 5 DPP, is known to be one of the first contaminants of the postpartum uterus and favor infection by other opportunistic bacteria, such as *F. necrophorum*, *A. pyogenes*, and *Bacteroides* sp. [3], [5]. Similarly, early studies have suggested that *A. pyogenes* is important for determining the prevalence of strictly anaerobic organisms, such as *F. necrophorum*, a relevant secondary invader in uterine infections [20]. However, in our study, sequences related to both *E. coli* and *A. pyogenes* were not significantly detected in any library (data not shown). Although we cannot completely rule out intrinsic bias of metagenomic studies, particularly DNA extraction and PCR bias, that might account for this observation, culture-independent diversity estimates are considerably less biased than culture-based methods, as the latter tend to target fast growing organisms and exclude fastidious microorganisms. It is important to restate that the sampling for the present study was performed at the 1–3, 8–10 and 34–36 DPP and our case definition for cows suffering from postpartum disorders was strict to animals that presented all classic clinical signs, as described elsewhere [4]. Our findings do not underscore the importance of *E. coli* and *A. pyogenes* in the pathogenesis of bovine metritis and endometritis, but we propose that other groups and, particularly, microorganisms so far uncultured or unidentified might have key role in the establishment of these pathogenesis. Although we do not have evidence, it is possible that some sequences detected in the uterine fluid may be fecal or vaginal contaminants. In addition, we would like to highlight that even known pathogens, as the ones detected in all the groups of cows in our study, might have minor role in uterine pathology.

We observed shift of the bacterial consortia in all the four distinct health statuses over the days of the study, suggesting that the bacterial community is dynamic. Besides the fact that the bacterial populations in samples from cows suffering from metritis presented seemingly chaotic shifts, overall, there seem to exist a defined bacterial core assembly for each health status, markedly evident at the 34–36 DPP. These types of observations raise the question of whether the microbiota plays an active causative role in defining the different levels or status of the diseases, or whether microbial imbalances is simply a consequence of the disease. For several human and other animal diseases, recent studies have shown that in fact the microbiota contributes to the disease [21]–[23]. It is likely that the same reasoning applies to reproductive disorders caused by bacteria in dairy cows, and, if confirmed, this correlation might influence or dictate therapeutic approaches for such diseases.

It is important to highlight that all dairy cows used in the present study were from a single commercial dairy farm and all samples were collected during a small time window of the year and generalization of these results to other production systems and time of the year should be cautiously considered. While we understand the limitations of our study, we reinforce that this is an effort to better characterize the microbiota in this infection and understand the possible ecology in this system. Thus, generalizations of the results observed here to other similar settings should be carefully considered. In fact, we have showed previously [12] that the farm environment, even more than the cow status, exerts more influence under richness and/or evenness in the uterine microbiota. Therefore, further research is needed to compare the environmental microbiota present in different farms as well as the immunological status of the cows and the individual immune response to pathogenic and opportunistic bacteria.

Our data showed that the profile of the microbiota is consistent with, but not conclusively demonstrative of, the health status of the cow. It is expected that many other host-dependent factors, such as age, immune status, and host-independent factors (e.g., the environment) contribute to cause the disease in postpartum cows. Finally, the mechanisms that link the microbial composition to these various health statuses remain elusive. The connection between bacterial and reproductive disorders in dairy cows is likely to be multifactorial and, indeed, future studies from our group will aim the biochemical and physiological potential of such microbiota as well as the effects of the microbiota on the host gene expression following calving.

In conclusion, the present study revealed a previously unappreciated fraction of the uterine bacterial composition in healthy dairy cows and in cows suffering from postpartum diseases. Overall, 1) bacterial succession occured, as suggested by the compositional shifts in the abundances of the major co-dominant groups, 2) the phylogenetic composition was affected by the health status of the cow, and 3) particularly at the 34–36 DPP, the composition seemed to be health-status-dependent. The data presented in this communication also provide an additional basis for culture-independent identification of bacterial groups predominant in bovine uterine fluid, which might be useful for practical applications. Subsequent research should address the functional proteome of the microbiome in the uterine environment, which will almost certainly clarify unknown aspects of the basic interactions of the core microbiota within the host and reveal potential entry points for effective therapeutic interventions of bovine reproductive disorders.

## Materials and Methods

### Animals and sampling

Uterine fluid samples were collected from April 2010 through June 2010 from postpartum Holstein dairy cows housed in a commercial dairy farm located near Ithaca, NY. Diagnosis of metritis and endometritis was according to Sheldon et al. (2006) [4]. Samples from each cow under investigation were collected three times during the study period; at 1–3 DPP, at 8–10 DPP, and at 34–36 DPP. Sixteen cows (4 healthy cows, 4 cows diagnosed with metritis, 4 cows diagnosed with metritis followed by endometritis, and 4 cows diagnosed with endometritis) were used for further investigation, resulting in a total of 48 samples that were processed independently, as described in the next sections. Two uterine sample collection methods were used; uterine swab for the first and second sampling and uterine lavage for the third sampling. Usually, by the 34–36 DPP, the uterus involutes significantly decreasing the diameter of the cervix and, consequently, the amount of uterine fluid. Thus, at this point, performing uterine lavage is a better way of sampling. In addition, uterine lavage was used at 34–36 DPP for the diagnosis of endometritis. Uterine swabs were collected according to the following procedure: cows were restrained and the perineum area was disinfected with 70% ethanol. Then, a sterile swab (Har-Vet™ McCullough Double-Guarded Uterine Culture Swab, Spring Valley, WI) covered by a sterile pipette (inside a plastic sheath) was introduced to the cranial vagina. To avoid vaginal contamination of the swab, the plastic sheet covered pipette was directed into cervix and inside the cervix the plastic sheath (first layer of protection) was ruptured and the pipette was then manipulated through the cervix into the uterus. Once inside the uterus, the swab was then advanced through the sealed plastic pipette (second layer of protection) exposing the sterile cotton swab to uterine secretion. The swab was pulled inside the pipette and removed while the pipette was maintained inside the uterus to avoid contamination by vaginal fluid. Swabs were introduced in transportation medium and were maintained at 4°C until processing. Uterine lavage samples were collected as described by Gilbert et al. (2005) [1]. Briefly, cows were restrained and the perineum area was disinfected with 70% ethanol. A plastic infusion pipette (inside a plastic sheath) was introduced to the cranial vagina. The infusion pipette was directed into the cervix and the plastic protection sheath was subsequently ruptured, avoiding potential contamination by vaginal fluid. Once inside the cervix, the clean pipette tip was manipulated through the cervix into the uterus. A total of 40 ml of sterile 0.9% sodium chloride solution (Baxter Co., Deerfield, IL) was injected into the uterus, agitated gently, and a sample of the fluid aspirated using a 20 ml syringe. The volume of recovered fluid ranged from 5 to 15 ml. Samples were transferred to sterile tubes and maintained in ice until processing. This project proposal was reviewed and approved by the Cornell University Institutional Animal Care and Use Committee (#2009-0001).

### DNA extraction

All samples were individually processed. For DNA extraction from swabs, the swabs were individually soaked in 30 ml of 0.1 M sodium phosphate buffer, pH 7.0, and incubated on ice for 1 h with occasional vigorous agitation to disperse the material. Subsequently, the samples were centrifuged for 30 min at 15,000×*g* at 4°C, the supernatants were discarded, and the pellets were suspended in 2 ml of 1×PBS buffer (137 mM NaCl, 2.7 mM KCl, 10 mM Na_2_HPO_4_, 1.76 mM KH_2_PO_4_) [pH 7.4]. For DNA extraction from uterine lavage, the sample was homogenized, an aliquot of 2 ml from each sample was transferred to sterile tubes and centrifuged for 30 min at 15,000×*g* at 4°C. The supernatants were discarded, and the pellets were suspended in 2 ml of 1×PBS buffer. Isolation of total metagenomic DNA was performed from 400 µL of the suspension by using a QIAamp DNA minikit (Qiagen, Valencia, CA, USA) according to the manufacturer's instructions for DNA purification from blood or body fluids. Some modifications, as addition of 400 µg of lysozyme and incubation for 12 h at 56°C, were included to maximize bacterial DNA extraction. Total DNA was eluted in 50 µl of sterile DNase/RNase-free water (Promega, Madison, WI, USA). The DNA concentration and purity were evaluated by optical density using a NanoDrop ND-1000 spectrophotometer (NanoDrop Technologies, Rockland, DE, USA) at wavelengths of 230, 260 and 280 nm. Additionally, integrity of the DNA was assessed by electrophoresis through a 0.8% (wt/vol) agarose gel, staining with 0.5 µg/ml ethidium bromide, and visualization with a MiniBIS Pro (DNR Bio-Imaging System Ltd., Jerusalem, Israel).

### PCR-DGGE

Analysis of the bacterial community by denaturing gradient gel electrophoresis (DGGE) was performed as described previously [12]. The 16S rRNA genes were individually amplified from each sample using two primer-pairs sets in two consecutive PCR. In a first round of PCR, using the primers 27F: 5′-AGAGTTTGATCMTGGCTCAG-3′ and 1522R: 5′-AAGGAGGTGATCCANCCRCA-3′ [24], bacterial-specific fragments were amplified and separately used as templates for a second PCR (nested PCR) primed by the conserved group-specific DGGE-primers [25] F984GC: 5′-AACGCGAAGAACCTTAC-3′, which contained a 40-bp GC-clamp [26] incorporated into the 5′ end, and R1378: 5′-CGGTGTGTACAAGGCCCGGGAACG-3′. Each 25-µl reactions contained 1× LongAmp *Taq* Reaction Buffer (60 mM Tris-SO_4_, pH 9.0; 20 mM (NH_4_)_2_SO_4_; 2 mM MgSO_4_, 3% glycerol; 0.06% NP-40; 0.05% Tween-20) (New England Biolabs), 0.2 mM dNTPs, 0.2 µM forward and reverse primers, 2.5 U LongAmp *Taq* DNA Polymerase (New England Biolabs), and approximately 50 ng of template DNA. Thermal cycling conditions for the first PCR were: initial denaturation for 2 min at 94°C, followed by 34 cycles of denaturation at 94°C for 30 sec, annealing at 58°C for 50 sec, extension at 72°C for 1 min, and a final extension at 72°C for 7 min. The cycle protocol for the nested PCR round was: initial denaturation for 5 min at 94°C, followed by 20 cycles of denaturation at 94°C for 1 min, annealing at 57°C for 1 min, extension at 72°C for 2 min, and a final extension at 72°C for 10 min. Reactions were performed in triplicate, pooled and purified using QIAquick PCR Purification kit (Qiagen). Appropriate PCR controls and blank were included and products were analyzed by electrophoresis in a 1.2% (wt/vol) agarose gel stained with 0.5 µg/ml ethidium bromide before DGGE analysis.

Profiling of the amplified 16S rRNA gene sequences produced by DGGE was conducted as described previously [27] using the DCode Universal Mutation Detection System apparatus (Bio-Rad, Hercules, CA, USA). The PCR products (∼2.5 µg) were loaded onto a 6% polyacrylamide (37.5∶1 acrylamide:*N,N′*-methylenebisacrylamide) (Sigma-Aldrich) parallel denaturing gradient gel in 1×TAE buffer (40 mM Tris [pH 8.0], 20 mM acetic acid, 1 mM EDTA [pH 8.0]) composed of 0.09% (vol/vol) TEMED (N,N,N′,N′-tetramethylenediamine), and 0.07% (wt/vol) ammonium persulfate. The denaturing gradient was optimized empirically to 40–58% urea/formamide (100% denaturant contained 7 M urea and 40% [vol/vol] deionized formamide). The electrophoresis was carried out in 1×TAE buffer at 50 V for 16 h at a constant temperature of 60°C. The DNA fragments were stained for 20 min in 1×TAE buffer with 1×SYBR Gold (Invitrogen). Images of the gels were obtained under UV light using an EC3 Imager imaging system (UVP). Sample lanes were analyzed using the BioNumerics version 6.0 (Applied Maths). The resulting patterns were compared to one another using the Dice similarity coefficient, and the matrix generated was clustered by the UPGMA method.

### PCR amplification of the V1–2 region of bacterial 16S rRNA genes

The 16S rRNA genes were individually amplified from each sample using a composite pair of primers containing a unique 10-base barcode (Table S1), which was used to tag the PCR products from each different sample. The forward primer used was 5′-**CGTATCGCCTCCCTCGCGCCATCAG**NNNNNNNNNNTC *AGAGTTTGATCCTGGCTCAG*-3′: the bold sequence is the GS FLX Titanium Primer A, and the italicized sequence is the universally conserved bacterial primer 27F. The reversed primer used was 5′- **CTATGCGCCTTGCCAGCCCGCTCAG**NNNNNNNNNNCA *TGCTGCCTCCCGTAGGAGT*-3′: the bold sequence is the GS FLX Titanium Primer B, and the italicized sequence is the broad-range bacterial primer 338R. The sequence NNNNNNNNNN, which is identical in the forward and reverse primer of each pair, designates the unique 10-base barcode used to tag each PCR product. A two-base linker sequence (underlined) was inserted between the barcode and the template-specific sequence to help diminish any effect the composite primer might have on the efficiency of the amplifications [28]. PCR were carried out in triplicates 20-µl reactions containing 0.3 µM forward and reverse primers, approximately 50 ng of template DNA and 1× HotStar Taq Plus Mix kit (Qiagen). A touchdown thermal cycling was used for amplification and consisted of initial denaturation at 95°C for 2 min, followed by 30 cycles of denaturation at 95°C for 30 sec, annealing (starting at 68°C and subsequently decreased by 2°C/2 cycles until it reached 58°C, temperature at which the 20 remaining cycles were performed) for 30 sec, extension at 72°C for 60 sec, and a final extension at 72°C for 7 min. Replicate amplicons were pooled, purified with Agencourt AMPure kit (Agencourt), and visualized by electrophoresis using 1.2% (wt/vol) agarose gels stained with 0.5 µg/ml ethidium bromide before sequencing. Blank controls, in which no DNA was added to the reaction, were performed similarly and, since these failed to produce visible PCR products, they were not analyzed further.

### Barcoded pyrosequencing of the bacterial 16S rRNA genes

Amplicons were quantified using the Quant-iT PicoGreen dsDNA Assay Kit (Invitrogen) and combined in equimolar ratios. Pyrosequencing of the samples was carried at the Cornell University Life Sciences Core Laboratories Center using Roche 454 GS-FLX System Titanium Chemistry.

### Pyrosequencing data analysis

Data quality control and most analysis were performed using the Quantitative Insights Into Microbial Ecology (QIIME) pipeline [29]. The raw reads obtained were assigned to their designated uterine sample using the split_library.py script, which also performs quality filtering based on length and quality of the reads. The parameters used were: minimum quality score = 25, minimum/maximum length = 200/1000, no ambiguous bases allowed and no mismatches allowed in the primer sequence. A maximum of 6 bp homopolimeric repeats were allowed. All the high-quality reads (average 344 bp without barcodes/two-base linker sequence/primers) obtained were aligned with PyNAST [30]. Following alignment, taxonomy assignment of the OTUs was performed; the clustering algorithm used was the furthest-neighbor and the degree of similarity between sequences defined based on 3% divergence. To estimate total bacterial diversity of DNA samples in a comparable manner, chimeras were removed using QIIME's ChimeraSlayer [31]. All the reads were also depleted of plastid, mitochondrial, and any non-16S bacterial reads. A total of 65,376 sequences were obtained after filtering and quality check. Taxonomy each OTU was assigned using the RDP classifier and the reference database used was the Greengenes database filtered at 97% identity. Briefly, data was compiled and relative percentage of each bacterial identification was determined for each individual sample. The outputs, based upon top hit designations, were compiled to generate percentage files at each taxonomic level. For the analysis, percentage values were calculated based on the total number of high-quality sequences for each cow. This analysis eliminates any interference of phyla that were highly abundant and allows the visualization of less abundant (or rare) groups. Another analysis was performed in which the sequences from the three different cows with the same health status and days postpartum (DPP) were combined to verify whether there was a general trend for cows presenting similar physiological conditions. In addition, the taxonomic analyses of the community was utilized to generate double hierarchal dendrograms, using the unweighted pair group method with arithmetic mean (UPGMA) and the Manhattan distance with no scaling, in the statistical software Number Cruncher Statistical System (NCSS) 2007. Unweighed and normalized Principal Component Analysis (PCoA) was performed to evaluate relatedness of samples. For this analysis, the high-quality sequences, prepared as described above, were used to generate an optimized phylogenetic tree that served as the input for UniFrac. Diversity indexes (Chao1 and Shannon) and rarefaction values were also generated by QIIME.

### Nucleotide sequence accession number

All sequences obtained were submitted to GenBank under accession numbers JX106521 to JX109583.

## Supporting Information

Table S1 **The 10-base multiplex identifier MID used in this study.**
(DOCX)
