# Supplementary material for: Diversity and Succession of Bacterial Communities in the Uterine Fluid of Postpartum Metritic, Endometritic and Healthy Dairy Cows
Source: PLoS One. 2012 Dec 27;7(12):e53048. doi: 10.1371/journal.pone.0053048 (PMC3531447; doi:10.1371/journal.pone.0053048)
Supplement: Table S1 — The 10-base multiplex identifier MID used in this study. (DOCX) [file pone.0053048.s001.docx]

Table S1. The 10-base multiplex identifier MID used in this study**.**

| MID number | Sequence (5'🡺3') |
| --- | --- |
| MID1 | **ACGAGTGCGT** |
| MID2 | **ACGCTCGACA** |
| MID3 | **AGACGCACTC** |
| MID4 | **AGCACTGTAG** |
| MID5 | **ATCAGACACG** |
| MID6 | **ATATCGCGAG** |
| MID7 | **CGTGTCTCTA** |
| MID8 | **CTCGCGTGTC** |
| MID10 | **TCTCTATGCG** |
| MID11 | **TGATACGTCT** |
| MID13 | **CATAGTAGTG** |
| MID14 | **CGAGAGATAC** |
| MID15 | **ATACGACGTA** |
| MID16 | **TCACGTACTA** |
| MID17 | **CGTCTAGTAC** |
| MID18 | **TCTACGTAGC** |
| MID19 | **TGTACTACTC** |
| MID20 | **ACGACTACAG** |
| MID21 | **CGTAGACTAG** |
| MID22 | **TACGAGTATG** |
| MID23 | **TACTCTCGTG** |
| MID24 | **TAGAGACGAG** |
| MID25 | **TCGTCGCTCG** |
| MID26 | **ACATACGCGT** |
| MID27 | **ACGCGAGTAT** |
| MID28 | **ACTACTATGT** |
| MID29 | **ACTGTACAGT** |
| MID30 | **AGACTATACT** |
| MID31 | **AGCGTCGTCT** |
| MID32 | **AGTACGCTAT** |
| MID33 | **ATAGAGTACT** |
| MID34 | **CACGCTACGT** |
| MID35 | **CAGTAGACGT** |
| MID36 | **CGACGTGACT** |
| MID37 | **TACACACACT** |
| MID38 | **TACACGTGAT** |
| MID39 | **TACAGATCGT** |
| MID40 | **TACGCTGTCT** |
| MID41 | **TAGTGTAGAT** |
| MID42 | **TCGATCACGT** |
| MID43 | **TCGCACTAGT** |
| MID44 | **TCTAGCGACT** |
| MID45 | **TCTATACTAT** |
| MID46 | **TGACGTATGT** |
| MID47 | **TGTGAGTAGT** |
| MID48 | **ACAGTATATA** |
| MID49 | **ACGCGATCGA** |
| MID50 | **ACTAGCAGTA** |

The MIDs were from the Technical Bulletin-Genome Sequencer FLX System (TB 0905 Roche MID Adaptors). (Available at: http://www.454.com/downloads/protocols/).
